# Increased Functional Connectivity During Emotional Face Processing in Children With Autism Spectrum Disorder

**DOI:** 10.3389/fnhum.2018.00408

**Published:** 2018-10-10

**Authors:** Kristina Safar, Simeon M. Wong, Rachel C. Leung, Benjamin T. Dunkley, Margot J. Taylor

**Affiliations:** ^1^Diagnostic Imaging, Hospital for Sick Children, Toronto, ON, Canada; ^2^Neurosciences and Mental Health Program, Research Institute, Hospital for Sick Children, Toronto, ON, Canada; ^3^University Health Network – Toronto Western Hospital, Toronto, ON, Canada; ^4^Department of Medical Imaging, University of Toronto, Toronto, ON, Canada

**Keywords:** ASD, emotional face processing, functional connectivity, magnetoencephalography, children

## Abstract

Individuals with autism spectrum disorder (ASD) demonstrate poor social functioning, which may be related to atypical emotional face processing. Altered functional connectivity among brain regions, particularly involving limbic structures may be implicated. The current magnetoencephalography (MEG) study investigated whole-brain functional connectivity of eight *a priori* identified brain regions during the implicit presentation of happy and angry faces in 20 7 to 10-year-old children with ASD and 22 typically developing controls. Findings revealed a network of increased alpha-band phase synchronization during the first 400 ms of happy face processing in children with ASD compared to controls. This network of increased alpha-band phase synchronization involved the left fusiform gyrus, right insula, and frontal regions critical for emotional face processing. In addition, greater connectivity strength of the left fusiform gyrus (maximal 85 to 208 ms) and right insula (maximal 73 to 270 ms) following happy face presentation in children with ASD compared to typically developing controls was found. These findings reflect altered neuronal communication in children with ASD only to happy faces during implicit emotional face processing.

## Introduction

An understanding of emotional information from faces is critical for successful social interaction. The primary indicator of autism spectrum disorder (ASD) is poor social functioning (American Psychiatric Association, 2013), which is thought to be related to atypical processing of emotional information from faces (see Harms et al., 2010). Findings from functional magnetic resonance imaging (fMRI) studies have suggested difficulties in early perceptual processing of emotional faces in ASD. For instance, individuals with ASD show reduced activation of the primary visual cortex and extrastriate areas, particularly the fusiform (Critchley et al., 2000; Hubl et al., 2003; Piggot et al., 2004; Wang et al., 2004; Deeley et al., 2007; Pelphrey et al., 2007), middle temporal (Critchley et al., 2000), and posterior superior temporal gyri (Pelphrey et al., 2005, 2007) in response to emotional faces. These regions are critical for identity recognition and face-specific information processing, including emotional faces (Haxby et al., 2000, 2002). Decreased activation of the amygdalae during emotional face processing has also been reported in those with ASD compared to typically developing controls, suggesting that the amygdalae may be differently involved in evaluating emotional stimuli in ASD (Pierce et al., 2001; Ashwin et al., 2007; Pelphrey et al., 2007; Corbett et al., 2009). Additionally, individuals with ASD show atypical frontal lobe activation when viewing emotional faces, particularly in the orbitofrontal, ventral prefrontal cortex, inferior frontal gyrus and anterior cingulate cortex (ACC, Dapretto et al., 2006; Ashwin et al., 2007; Loveland et al., 2008). Reduced bilateral insulae activation has been observed during introspection of emotional states (Silani et al., 2008) and activation of the right insula is reduced during the presentation of emotional faces in ASD (Di Martino et al., 2009). The insulae and ACC are key nodes of the salience network, which is pertinent in the evaluation of one’s own and others’ emotional states, emotional judgment and empathy (Di Martino et al., 2009; Uddin and Menon, 2009; Menon and Uddin, 2010).

Recent research has focused on examining the interconnectedness of networks fundamental for neuronal communication in ASD (see Just et al., 2012, for review). A growing body of research indicates that individuals with autism exhibit widespread abnormal functional connectivity (see Uddin et al., 2013b; Kana et al., 2014; Ha et al., 2015, for reviews), however, only a handful of studies have investigated functional connectivity of brain regions implicated in face and emotional face processing in ASD (Kleinhans et al., 2008; Monk et al., 2010; Ebisch et al., 2011; Rudie et al., 2011; Gotts et al., 2012; Kana et al., 2016). The majority of task-based functional connectivity MRI (fcMRI) studies assessing face and emotional face processing have reported decreased connectivity in adults with ASD compared to controls (Kleinhans et al., 2008; Rudie et al., 2011; Kana et al., 2016), although one study found mixed results (Monk et al., 2010). Kana et al. (2016) reported widespread reduced functional connectivity of the medial prefrontal cortex with the bilateral amygdalae, superior temporal gyri, inferior parietal lobules, and fusiform gyri during implicit processing of emotional scenarios in adults with ASD.

As brain functional connectivity is dynamic, neurophysiological techniques that provide better time resolution than fMRI can complement our understanding of connectivity differences between those with and without ASD. However, only a few studies using electroencephalography (EEG) or magnetoencephalography (MEG) have examined whether functional connectivity is disrupted in social cognitive tasks in ASD (Khan et al., 2013; Leung et al., 2014; Yeung et al., 2014; Mennella et al., 2017). Using MEG, Khan et al. (2013) found reduced phase – amplitude coupling between alpha and gamma oscillations in the fusiform during face processing in adolescents with ASD compared to typically developing controls. In addition, decreased long-range alpha-band functional connectivity was seen between the fusiform and left precuneus, left inferior frontal gyrus and left ACC to emotional faces in the adolescents with ASD. A more recent MEG study (Leung et al., 2014) examined theta, alpha and beta-band phase synchrony during the implicit processing of emotional faces in adolescents with ASD. Findings revealed reduced beta-band phase synchrony 0–400 ms following angry face presentation among a network of brain regions implicated in emotional face processing in the adolescents with ASD compared to typically developing controls. Particularly, the right insula was a hub region of decreased functional connectivity strength within this network. Using MEG and the same task, Mennella et al. (2017) also found reduced beta-band phase synchronization in adults with ASD. Specifically, hypoconnected regions important for emotional face processing included the left amygdala, left insula, and striatum. MEG is advantageous as it captures direct and “real-time” neural activity and offers a combination of high spatial resolution (Hari and Puce, 2017) and excellent, millisecond temporal resolution (Hansen et al., 2010). To our knowledge, no studies have used MEG to investigate functional connectivity during emotional face processing in children with ASD, which is critical to elucidate the developmental trajectory of neural connections to faces in this neurodevelopmental disorder.

Using MEG, the current study examined whole-brain functional connectivity of eight *a priori* regions of interest (ROIs) during implicit emotional face processing in children with ASD compared to typical controls. As ROIs we selected the bilateral insulae and bilateral ACC, regions that comprise the salience network, the bilateral fusiform gyri and amygdalae from the Automated Anatomical Labeling (AAL) atlas (Tzourio-Mazoyer et al., 2002). These ROIs were selected as they are known to be involved in emotional face processing, and in other studies were sensitive to atypical functional connectivity in ASD (Seeley et al., 2007; Kleinhans et al., 2008; Di Martino et al., 2009; Ebisch et al., 2011; Barrett and Satpute, 2013; Khan et al., 2013; Leung et al., 2014; Uddin, 2014; Mennella et al., 2017). To determine whole-brain functional networks involving these *a priori* ROIs during implicit emotional face processing, we examined pairwise phase synchrony between these eight AAL ROIs and all 90 subcortical and cortical AAL regions over the whole brain. We modeled the design of the present study on Leung et al. (2014) described above. Based on Leung et al. (2014), we hypothesized that children with ASD would show decreased beta-band interregional functional connectivity involving the eight *a priori* ROIs during implicit angry face processing compared to typically developing controls.

## Materials and Methods

### Participants

Twenty children with ASD (*M* age = 9.4 years, *SD* = 1.38, range: 7.0–10.7 years, 15 boys, 17 right-handed) and 22 age equivalent typically developing children (*M* age = 8.6 years, *SD* = 1.34, range: 7.0–10.8 years, 18 boys, 20 right-handed) participated. Exclusion criteria included comorbid neurological or neurodevelopment disorder, brain injury, uncorrected visual impairment, color blindness, IQ ≤ 55 or ≥ 145 (±3 standard deviations from the population mean), and/or language skills inadequate for completion of the tasks. Four children with ASD were on medication, including Biphentin (*n* = 2), Strattera (*n* = 1), and Dexodrin (*n* = 1). The Wechsler Abbreviated Scale of Intelligence (WASI; Wechsler, 1999) was used to assess IQ. Although mean IQ in both groups was within the normal range, children with ASD demonstrated lower Verbal and Performance subtest WASI IQ scores (*M* = 101.35, *SD* = 17.21), compared to typically developing controls (*M* = 113.29, *SD* = 13.98), *t*(39) = −2.44, *p* = 0.019, *d* = 0.76. For children in the ASD group, a diagnosis was confirmed by expert clinical judgment, medical diagnostic reports and the Autism Diagnostic Observation Schedule (ADOS-G and ADOS-2 module 3, Lord et al., 2000, 2012). The total mean ADOS calibrated severity score for children in the ASD group equaled 7.05 (*SD* = 1.65). The protocol was approved by the Hospital for Sick Children Research Ethics Board. All parents gave written informed consent and all child subjects gave verbal assent in accordance with the Declaration of Helsinki.

### Emotional Faces Task and MEG Data Acquisition

The emotional faces task used in the current study is described in Leung et al. (2014). Color photographs of 25 different faces (13 males) each expressing happy or angry emotions with validity ratings of at least 80% accuracy were drawn from the NimStim Face Stimulus Set (Tottenham et al., 2009). Scrambled pattern face counterparts corresponding to each face were generated by applying a mosaic filter to the faces (15 pixels per cell), which were then divided into 64 square tiles, shuffled and Gaussian blurred (10.0 pixels) using Photoshop (Adobe Systems, Inc., San Jose, CA, United States). Each scrambled pattern corresponded to the face with which it was paired. Thus, these scrambled patterns matched the selected faces on luminosity and color, to preserve low-level visual properties of the faces.

**FIGURE 1:**
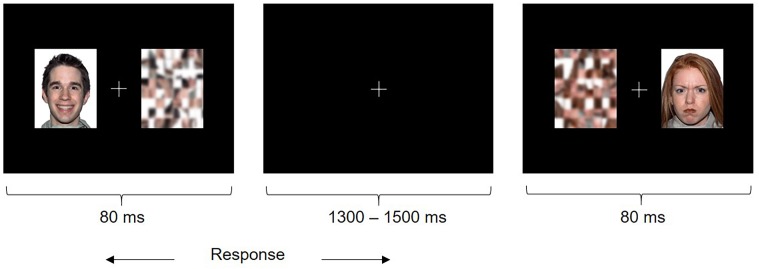
Emotional Faces Task. Participants saw a happy or angry face and scrambled pattern (target) simultaneously on either side of a fixation cross. Participants were instructed to attend to the fixation cross and indicate the left or right position of the target as quickly as possible using a button press.

Each trial consisted of a face (happy or angry, which was a distractor) and the corresponding scrambled pattern (which was the target) presented simultaneously for 80 ms on either side of a central fixation cross (**Figure 1**). Children were instructed to fixate the central cross and quickly indicate the position of the target by pressing a button (left or right) and ignore the distracter. The emotional faces were behaviorally irrelevant to the task, which allowed us to probe implicit emotional face processing that may be particularly impaired in individuals with ASD relative to explicit emotional face processing, as it is automatic, rapid and subconscious in nature (Frith, 2004). The duration of stimuli was 80 ms followed by an inter-stimulus interval jittered between 1300 and 1500 ms. Participants saw 50 trials per face type presented twice each in the left and right hemifields, in randomized order (200 happy and angry trials in total). Stimuli were presented at a viewing distance of approximately 75 cm and subtended approximately 6.9 degrees of visual angle. The task was presented using Presentation^®^ (Neurobehavioral Systems).

MEG data were acquired with a 151 channel CTF system in a magnetically shielded room in the supine position. Continuous head localization was performed with fiducial coils at the left and right pre-auricular points and the nasion. Data were continuously sampled at 600 Hz with an online 150 Hz antialiasing filter. After MEG data acquisition, fiducial coils were substituted for radio-opaque markers for MRI co-registration. A T1-weighted MRI scan (3D SAG MPRAGE, GRAPPA = 2, TR/TE/FA = 2300 ms/2.96 ms/90°, FOV = 28.8 cm × 19.2 cm, 240 × 256 matrix, 192 slices, slice thickness = 1.0 mm isotropic voxels) was obtained for each child on a 3T MRI scanner (MAGNETOM Tim Trio, Siemens AG, Erlangen, Germany) with a 12-channel head coil.

### MEG Source Reconstruction

Magnetoencephalography data were co-registered with each individual’s MRI using the fiducial markers. The data were then segregated into −500 to 1500 ms epochs by face type (angry or happy). Epoched data trials were excluded from analysis if head movement exceeded 10 mm from the median head position in each trial, a threshold commonly used with MEG in examining pediatric populations (Pang, 2011; Taylor et al., 2011; Doesburg et al., 2013). In the remaining trials, there were no significant group differences in head movement for happy faces [*t*(40) = −0.014, *p* = 0.989, *d* = 0.004] or angry faces [*t*(40) = 0.074, *p* = 0.942, *d* = 0.023]. All trials were visually inspected and if required excluded due to artifact such as eye movement, blinks, etc. In addition, by applying a spatial filter, MEG beamforming is effective at attenuating artifacts (Muthukumaraswamy, 2013). Mann–Whitney tests calculated due to non-normality of the distributions revealed that the number of happy trials included (ASD: *Mdn* = 87.5, controls: *Mdn* = 95) did not differ between groups (*U* = 281.5, *z* = 1.55, *p* = 0.12), nor did the number of angry trials included (ASD: *Mdn* = 91, controls: *Mdn* = 93; *U* = 267.5, *z* = 1.19, *p* = 0.231). The FieldTrip software toolbox (git commit 4c12371; Oostenveld et al., 2011) was used for MEG data processing. A single shell head model was constructed from each child’s individual MRI and was normalized onto a standard MRI brain template (ICBM 152; Fonov et al., 2009, 2011). The data were filtered offline with a 4th order two-pass Butterworth bandpass 1–150 Hz filter. The coordinates for the center-of-mass of each of the first 90 sources (seeds) representing all subcortical and cortical brain structures of the AAL atlas were unwarped from the standard MRI brain template space into equivalent locations for each child’s headspace (Tzourio-Mazoyer et al., 2002). A linearly constrained minimum variance (LCMV) beamformer (Van Veen et al., 1997) reconstructed the broadband time-series of brain activity for these source locations for each trial. The beamformer estimates activity at a given source of interest while reducing activity and noise from other neural and extracranial sources of no interest by applying a spatial filter (see Doesburg et al., 2013). The LCMV beamformer was used to compute a single common spatial filter for each subject based on the covariance of all selected trials. Five percent Tikhonov regularization was applied to increase the robustness of the inverse solution and the Neural Activity Index was computed (Van Veen et al., 1997) to account for the center-of-head bias due to correlated noise.

### Functional Connectivity: Phase Synchronization

Magnetoencephalography data were filtered into theta (4–7 Hz), alpha (8–14 Hz), beta (15–30 Hz), and gamma (30–80 Hz) canonical frequency-bands using a two-pass FIR filter. The delta frequency-band was not analyzed because it nears the noise floor of the MEG, and a task trial does not provide enough data to adequately characterize delta oscillations. The filters were designed based on the Hamming window using the fir1 function in MATLAB (The Mathworks Inc., 2016) software. We filtered the data into frequency-bands after beamforming as the covariance matrices derived from signals are different for each frequency, which would result in signals from the same target voxel in each frequency-band being variably localized from different areas. The Hilbert Transform was used to compute instantaneous time series of phase values for each source and frequency-band. To assess synchrony of phase oscillations between brain regions we used the phase-lag index (PLI) based on Stam et al. (2007). The PLI estimates the phase synchrony between time series by characterizing the consistency by which the two time series lead or lag relative to each other while ignoring zero phase lag. This measure attenuates artificial phase synchrony by eliminating near or at zero phase lag between sources (Stam et al., 2007). The PLI was calculated between the eight selected AAL source locations (left and right insulae, ACC, amygdalae, and fusiform gyri; see **Supplementary Table 1** for a list of center-of-mass coordinates for these regions) and each of the other 90 subcortical and cortical AAL regions over the whole brain, across trials. This generated a 90 × 8 adjacency matrix for each time point within each frequency-band, for each face type, and participant.

An active time window of phase synchrony between 0 and 400 ms following stimulus onset for each participant was selected for statistical analysis. This active window was based on Leung et al. (2014), who found differences in interregional phase synchronization 0–400 ms after stimulus presentation, and confirmed by visual examination of task-based changes in PLI across the time series. PLI values at each time point within this active window were z-scored relative to a baseline interval of −500 to 0 ms prior to stimulus onset. For each participant normalized PLI values from each time point were averaged within the active window.

### Statistical Analysis

The Network Based Statistic (NBS) toolbox was used to determine statistical significance of phase synchronization within- and between-groups. NBS is a non-parametric method to identify significant differences in networks of connections between groups while controlling for family wise error rate (FWER; Zalesky et al., 2010, 2012). Significant differences in network connectivity are tested by first applying a *t*-test to the PLI values (z-scored in this case) at every connection of the 90 × 8 adjacency matrix, which yields a *t*-value for each connection. The *t*-values are then thresholded, and connections exceeding this threshold (referred to as suprathreshold connections) that comprise a component (a contiguous cluster of suprathreshold connections) may be identified and subjected to permutation testing at the network level. We chose our *t*-statistical thresholds based on Zalesky et al. (2010), in which we used an iterative adaptive threshold specific to the data distributions for optimal NBS performance; thus we set the *t*-test statistical threshold for within-group comparisons analysis to *t* = 1.8 and for between-group comparisons analysis to *t* = 2.75. For each permutation (5,000 permutations in the current study), group membership is randomly shuffled and the largest component observed is recorded to create a null distribution. The null distribution is then compared to the size of the originally identified, empirically derived, component to assess statistical significance and a FWER corrected *p*-value is assigned accordingly (Zalesky et al., 2010, 2012).

## Results

### Behavioral

No significant group difference in reaction time (ms) to happy faces [*t*(40) = −0.147, *p* = 0.884], or angry faces [*t*(40) = −0.013, *p* = 0.99] was seen. For accuracy, Mann–Whitney tests were calculated due to non-normality of the distributions; no significant group difference was found to happy faces (*U* = 209, *z* = −0.277, *p* = 0.781), or angry faces (*U* = 209, *z* = −0.278, *p* = 0.788). No between-group differences at the behavioral level is not surprising since the task was implicit and undemanding, which assures that the group differences in functional connectivity cannot be attributable to poorer task performance by the children with ASD.

### Within-Group Alpha-Band

For the within-group analysis, the active window (0–400 ms) was compared to baseline (−500 to 0 ms) for both groups of children, for happy and angry faces, separately. Findings revealed significantly increased phase synchronization for children with and without ASD in the alpha frequency-band, 0 to 400 ms following stimulus onset during the processing of happy and angry faces. Networks indicating significantly increased phase synchronization encompassed interregional connections among all eight ROIs and other widespread brain regions for angry faces in children with ASD and typically developing controls, and for happy faces in children with ASD. For happy faces in controls, a network involving interregional connections among seven of the eight ROIs (left and right insulae, left ACC, left and right amygdalae and left and right fusiform gyri) and other brain regions was found. In controls, the network involved 82 edges and 59 nodes for angry faces (*p_corr_* < 0.0002 ^1^), and 45 edges and 39 nodes for happy faces (*p_corr_* = 0.036). In children with ASD, the network involved 101 edges and 70 nodes for angry faces (*p_corr_* < 0.0002), and 95 edges and 65 nodes for happy faces (*p_corr_* < 0.0002).

### Between-Group: Increased Alpha-Band Phase Synchrony in ASD

**FIGURE 2:**
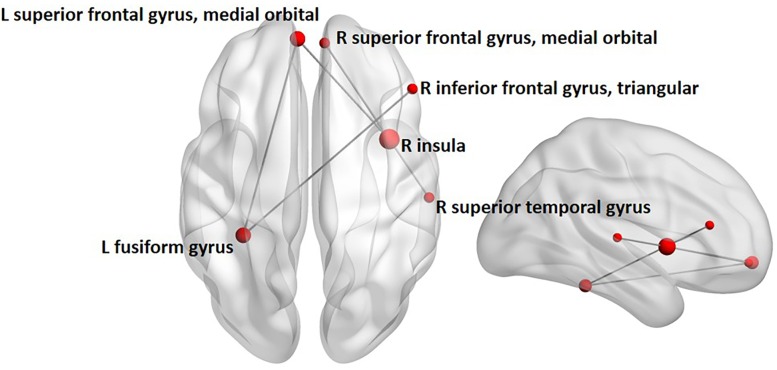
Between-group analysis of alpha-band phase synchronization during happy face processing in children with ASD compared to controls. Network indicating increased alpha-band phase synchronization in children with ASD relative to controls 0 to 400 ms following the presentation of happy faces. Node size is modulated by the degree of mean group difference in connectivity strength.

Between-group contrasts were conducted for each frequency-band (i.e., theta, alpha, beta, and gamma) and emotion condition (i.e., angry and happy). Findings revealed increased alpha-band phase synchronization during happy face processing, 0–400 ms following stimulus onset in children with ASD, compared to controls (**Figure 2**). The network comprised five edges and six nodes (*p_corr_* = 0.02), involving connections between the left fusiform and left superior frontal gyrus, medial orbital part, and right inferior frontal gyrus, triangular part, and connections between the right insula and right superior temporal gyrus, right superior frontal gyrus, medial orbital part, and left superior frontal gyrus, medial orbital part. No significant differences between groups were found for angry faces in the alpha frequency-band. No significant group differences were found in the theta, beta, and gamma frequency-bands for angry or happy faces (see **Supplementary Table 2** for a summary of all between-group network contrasts).

### Connectivity Strength Analysis

**FIGURE 3:**
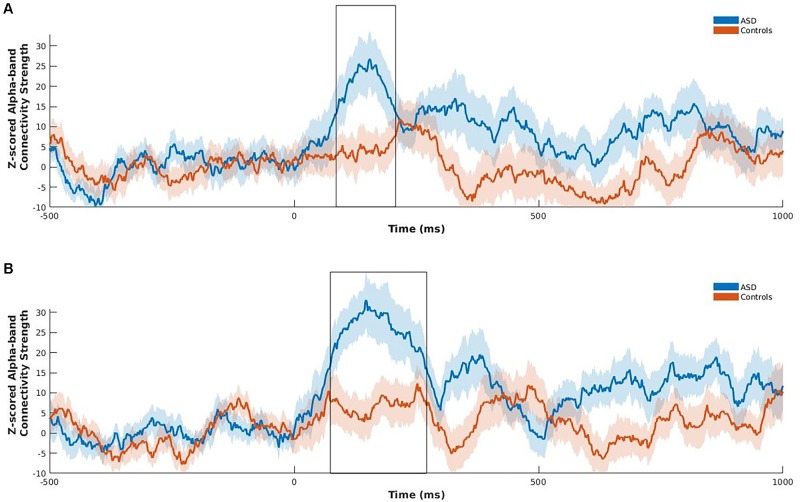
Z-scored alpha-band connectivity strength analysis during happy face processing in children with ASD compared to controls. Children with ASD showed increased z-scored alpha-band connectivity strength maximal between 85 and 208 ms for the left fusiform **(A)**, and 73 to 270 ms for the right insula **(B)** following onset of happy faces relative to controls. Shading represents standard error of the mean.

Following the NBS analysis we computed connectivity strength of the left fusiform and right insula, as only these two regions of the eight *a priori* ROIs were significantly hyperconnected in children with ASD in the between-group contrast, and are known to play a key role in emotional face processing and atypically functionally connected in ASD (Kleinhans et al., 2008; Khan et al., 2013; Leung et al., 2014; Mennella et al., 2017). Strength was computed at each time point across the active window, within the alpha frequency-band for happy faces for these two regions only. Strength is the sum of a specific region’s connections to all other brain regions and is an indication of that region’s importance in a network (Rubinov and Sporns, 2010). Permutation testing was used to investigate group differences using 5000 permutations. Benjamini–Hochberg adjusted *p*-values were used to correct for multiple comparisons (*p*_BH_ < 0.05; Benjamini and Hochberg, 1995). Positive z-scored PLI values indicate greater phase synchrony relative to baseline, whereas zero or negative values indicate no change or decreased phase synchrony relative to baseline, respectively. Significantly increased z-scored alpha-band connectivity strength was maximal between 85 and 208 ms following stimulus onset for the left fusiform gyrus (*p*_BH_ = 0.02), and between 73 and 270 ms for the right insula (*p*_BH =_ 0.012) in children with ASD relative to controls (**Figure 3**).

### Correlation Between Strength and ADOS Calibrated Severity Score

**FIGURE 4:**
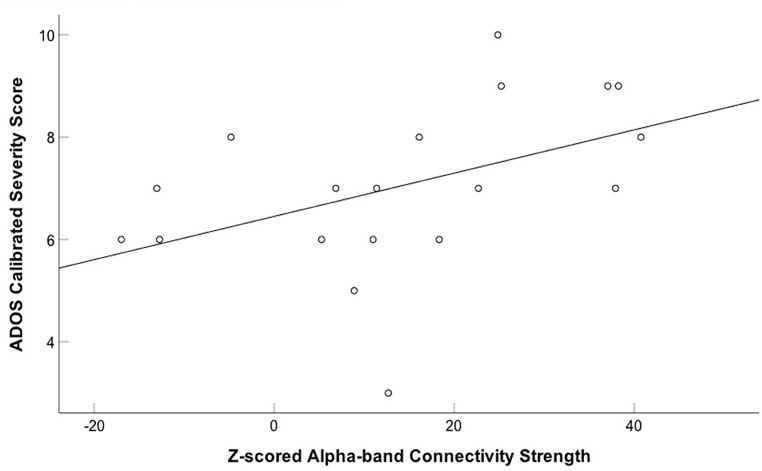
Positive correlation between z-scored alpha-band connectivity strength of the left fusiform gyrus to happy faces and ADOS calibrated severity score in children with ASD. A positive correlation between z-scored alpha-band connectivity strength of the left fusiform gyrus to happy faces and ADOS calibrated severity score was found in the children with ASD.

We also correlated connectivity strength of the left fusiform and right insula with the ADOS calibrated severity score in children with ASD. We found a significantly positive correlation between z-scored alpha-band connectivity strength of the left fusiform gyrus and ADOS calibrated severity score (*r* = 0.455, *p* = 0.05; uncorrected) during the first 400 ms following happy face presentation in the children with ASD (**Figure 4**). After correction for multiple comparisons this result was no longer significant (*p*_BH =_ 0.1). No significant correlation was found between z-scored alpha-band connectivity strength of the right insula and ADOS calibrated severity score (*r* = 0.225, *p* = 0.354).

## Discussion

The current study is the first to address whether whole-brain phase synchronization of eight well-established emotional face processing regions is disrupted in children with ASD during implicit emotional face processing using MEG. Contrary to our hypothesis, results indicated increased alpha-band phase synchronization involving the fusiform gyrus and insula during the first 400 ms of implicit happy face processing in children with ASD compared to age-matched controls. Specifically, this network of increased alpha-band phase synchronization in ASD involved connections between the left fusiform and frontal regions including the left superior frontal gyrus, medial orbital part, and right inferior frontal gyrus, as well as connections between the right insula, the right superior temporal gyrus and orbitofrontal areas. Group differences in strength were found in the left fusiform gyrus and right insula. Thus, in children with ASD, these two regions were more strongly connected to all other brain regions than in typically developing controls. Furthermore, the ADOS calibrated severity score was positively correlated (uncorrected) with connectivity strength of the left fusiform gyrus, suggesting an association between greater connectivity strength and higher ASD symptomology.

Phase synchrony among brain regions underlies neuronal communication (Fries, 2005). The current findings suggest atypical communication among brain areas critically implicated in early perceptual processing of emotional faces, particularly the fusiform and superior temporal gyri (Schultz, 2005), and higher-level areas involved in extracting and evaluating affective information, such as the inferior frontal gyrus, orbitofrontal cortex, and insula (Nakamura et al., 1999; Schultz et al., 2000; Craig, 2002; Rolls, 2004) in children with ASD. Increased functional connectivity between the right insula – a part of the salience network – and other emotion processing regions may contribute to difficulties in assessment of salient emotional stimuli, such as facial expressions in children with ASD (see Menon and Uddin, 2010). Furthermore, greater alpha-band connectivity strength maximal 85 to 208 ms in the left fusiform, and 73 to 270 ms in the right insula during happy face processing is consistent with previous MEG studies showing activation of the bilateral fusiform gyri and right insula to emotional faces overlapping with these time windows (Chen et al., 2009; Hung et al., 2010).

Our findings of atypical functional connectivity in these brain areas in ASD are aligned with previous research examining emotional face processing (Leung et al., 2014; Kana et al., 2016; Mennella et al., 2017). However, these studies have reported decreased functional connectivity, rather than increased in ASD. One explanation for this discrepancy may be the age of participants we studied. Recently, a developmental model has been proposed to account for differences in hypo- vs. hyper-connectivity in ASD marked by the onset of puberty, indicating adolescence, which initiates considerable structural and functional developmental changes in the brain that temporally corresponds with changes in reported patterns of functional connectivity in the literature (Uddin et al., 2013b; Mamashli et al., 2018). For instance, Mamashli et al. (2018) have recently found that age (7–21 years) and local and long-range functional connectivity are oppositely correlated in ASD compared to controls in response to emotional faces; such that functional connectivity metrics decreased with age in ASD, while increased with age in controls. Accordingly, Picci and Scherf (2015) and Picci et al. (2016) have proposed that adolescence may denote a period of susceptibility for individuals with ASD due to biological factors and environmental pressures that can trigger extensive changes in both behavioral and neural functioning. Consistent with this developmental approach, young children with ASD tend to demonstrate hyperconnectivity of extensive intrinsic networks in fMRI compared to typically developing controls, unlike adolescents and adults with ASD (Di Martino et al., 2011; Supekar et al., 2013; Uddin et al., 2013a). For example, Uddin et al. (2013a) observed greater functional connectivity of the salience network (the ACC and bilateral insulae) in children with ASD during resting-state. In particular, individual participant maps of the salience network discriminated children with ASD from controls with greater classification accuracy (78%) than other networks. The authors suggested that increased functional connectivity of the salience network might be a biomarker of atypical evaluation and processing of emotional information in children with ASD. Similarly using fcMRI, Supekar et al. (2013) examined whole-brain intrinsic functional connectivity in a large sample of children with and without ASD. Increased widespread functional connectivity across both long- and short-range connections was reported in children with ASD. Increased functional connectivity was correlated with greater regional amplitudes of low-frequency fMRI signal fluctuations indicating atypical local neural circuit functioning. In an EEG study, high-risk infant siblings later diagnosed with ASD also demonstrated greater alpha-band functional connectivity in response to short social and non-social videos recorded over fronto-central scalp regions (Orekhova et al., 2014). Thus, our finding of increased phase synchronization in response to implicit emotional faces in children with ASD is consistent with this body of literature.

Why might children with ASD show patterns of increased functional connectivity to happy faces? It is possible that increased connectivity may reflect an abnormal trajectory of early functional brain development (Uddin et al., 2013a; Orekhova et al., 2014). Atypical critical periods of synaptic plasticity may play an important role in greater functional connectivity in children with ASD (LeBlanc and Fagiolini, 2011; Uddin et al., 2013a), as a balance of cortical excitatory and inhibitory neurotransmission is fundamental for the onset and closure of critical periods of neural plasticity (LeBlanc and Fagiolini, 2011). In ASD, dysregulation of the manifestation and timing of critical periods may occur due to elevated levels of excitation or decreased inhibition. This imbalance results in a “hyper-excitable cortex” and greater levels of plasticity giving rise to noisy and unstable processing in the brain (Rubenstein and Merzenich, 2003; LeBlanc and Fagiolini, 2011). Importantly, hyperexcitability has been linked to and suggested to contribute to whole-brain increased functional connectivity in children with ASD (Supekar et al., 2013). It is possible that this increased functional connectivity may be indicative of an elevated ratio of cortical excitatory vs. inhibitory neurotransmission in children with ASD. Results of our study suggest that this may be particularly evident in a network involved in implicit happy face processing, perhaps underlying noisy and disrupted communication. Our finding of greater connectivity strength of the left fusiform gyrus correlated with increased ASD symptomology in children with ASD (prior to correction for multiple comparisons), as well as those of previous studies that have found an association between hyperconnectivity and increased symptom severity in ASD (i.e., greater social impairment) supports this possibility (Kleinhans et al., 2008; Keown et al., 2013).

The current findings of increased phase synchronization in response to implicit happy facial expressions in children with ASD may alternatively reflect a network that is more responsive to happy faces in children with ASD compared to typically developing controls. Monk et al. (2010) observed increased functional connectivity between the right amygdala and ventromedial prefrontal cortex in response to happy faces in adults with ASD (although overall networks of both increased and decreased functional connectivity were reported), suggesting that this particular network is more responsive to happy faces in ASD compared to typically developing controls. Happy is the first facial expression to be accurately recognized in typically developing children (Markham and Adams, 1992; Gao and Maurer, 2010), young children show a positivity bias compared to adolescents (Boseovski and Lee, 2008) and happy faces are more salient to young children than angry faces (Todd et al., 2011). In children with ASD, happy face processing is relatively spared compared to negative emotions such as anger, fear and sadness, perhaps due to increased experience with and familiarity of happy faces (Farran et al., 2011). Thus, our findings of hyperconnectivity during happy face processing in children with ASD may reflect an amplified network response, which may be interpreted as spared processing of happy over angry faces in early childhood. Happy face processing may decrease sooner in typically developing children, as with age their skills increase with other emotions. Future research correlating emotion recognition accuracy, familiarity and intensity measures with phase synchronization during emotional face processing are necessary to evaluate this possibility.

Unlike adolescents (Leung et al., 2014), children with ASD show atypical phase synchronization in the alpha frequency-band. The effects seen in the alpha-band in the children, but beta-band in adolescents are consistent with other reports of effects emerging in lower frequency-bands in younger, particularly clinical, cohorts (e.g., Doesburg et al., 2013). Furthermore, alpha-band functional connectivity is known to be implicated in visual perception of relevant stimuli in typically developing individuals (Freunberger et al., 2008; Hanslmayr et al., 2011; Palva and Palva, 2011). In an EEG study, alpha-band phase synchronization between frontal and posterior regions was increased during the perception and identification of a meaningful object, which was suggested to reflect retrieval of semantic information via top-down processing (Freunberger et al., 2008). Thus, increased alpha-band phase synchrony in the present study might indicate abnormal perceptual or higher-level processing of the happy faces.

## Conclusion

We investigated functional connectivity during implicit emotional face processing in children with and without ASD using MEG. Our findings revealed increased alpha-band phase synchronization during happy face processing in children with ASD and increased connectivity strength of the right insula and left fusiform gyrus. These results suggest atypical communication among brain regions critical for the perception and higher-level processing of emotional faces. Given findings in adults and adolescents (Leung et al., 2014; Mennella et al., 2017), this pattern of results further supports an altered neurodevelopmental trajectory of emotional face processing in ASD, which has implications for establishing earlier targeted interventions that may better emotion processing abilities and broader socio-emotional functioning in this population. The current study establishes a necessary foundation for future works to replicate findings with a larger sample size, particularly the association between connectivity strength and ASD symptomology may be further examined, as well as an expanded age range of participants to directly investigate age-related changes in neural networks to emotional faces across development – an important next step.

## Author Contributions

KS contributed to data analysis and interpretation, drafting of the manuscript, and approved the manuscript for publication. SW contributed to data analysis, revised the manuscript, and approved the manuscript for publication. RL contributed to task design and data acquisition, revised the manuscript, and approved it for publication. BD contributed to data analysis and interpretation, revised the manuscript, and approved it for publication. MT contributed to the design of the task and interpretation of data, revised the manuscript, and approved it for publication.

## Conflict of Interest Statement

The authors declare that the research was conducted in the absence of any commercial or financial relationships that could be construed as a potential conflict of interest.

## Footnotes

1 *p* < 0.0002 indicates that less than one out of the 5000 permutations computed resulted in a network that was greater than the unpermuted network.
