# Supplementary material for: Increased Functional Connectivity During Emotional Face Processing in Children With Autism Spectrum Disorder
Source: Front Hum Neurosci. 2018 Oct 10;12:408. doi: 10.3389/fnhum.2018.00408 (PMC6191493; doi:10.3389/fnhum.2018.00408)
Supplement: Supplementary file 1 [file Table_1.DOCX]

Supplementary Material

Table 1. List of AAL centre-of-mass coordinates for the eight *a priori* regions of interest (ROIs).

| Regions of interest (ROIs) | x y z |
| --- | --- |
| Insula L | -36 7 3 |
| Insula R | 38 6 2 |
| ACC L | -5 35 14 |
| ACC R | 7 37 16 |
| Amygdala L | -24 -1 -17 |
| Amygdala R | 26 1 -18 |
| Fusiform L | -32 -40 -20 |
| Fusiform R | 33 -39 -20 |
