# Supplementary material for: Increased Functional Connectivity During Emotional Face Processing in Children With Autism Spectrum Disorder
Source: Front Hum Neurosci. 2018 Oct 10;12:408. doi: 10.3389/fnhum.2018.00408 (PMC6191493; doi:10.3389/fnhum.2018.00408)
Supplement: Supplementary file 2 [file Table_2.DOCX]

Supplementary Material

Table 2. Summary of all between-group network contrasts, 0–400 ms, t = 2.75.

| Frequency-band | Condition | ASD > Controls (*p_corr_*) | ASD < Controls (*p_corr_*) |
| --- | --- | --- | --- |
| Theta | Angry | 0.905 | 0.883 |
|  | Happy | 0.898 | 0.882 |
| Alpha | Angry | 0.910 | 0.888 |
|  | Happy | 0.020* | 0.884 |
| Beta | Angry | 0.438 | 0.412 |
|  | Happy | 0.419 | 0.120 |
| Gamma | Angry | 0.932 | 0.928 |
|  | Happy | 0.933 | 0.925 |

*p < 0.05, one-tailed
